# Positive Psychology in Non‐WEIRD Countries: Contexts, Challenges, and Opportunities

**DOI:** 10.1002/pchj.70112

**Published:** 2026-07-26

**Authors:** Yanhui Mao, Frederick T. L. Leong

**Affiliations:** ^1^ Institute of Applied Psychology, Psychological Research and Counseling Center, Southwest Jiaotong University Chengdu China; ^2^ Division of Applied Psychology, School of Humanities and Social Science Chinese University of Hong Kong Shenzhen China

Positive psychology, broadly defined as the scientific study of the strengths, virtues, and conditions that enable individuals and communities to thrive, has expanded rapidly since its formal articulation at the turn of the millennium (Seligman and Csikszentmihalyi 2000). Yet its cumulative knowledge base has been built overwhelmingly within WEIRD (Western, Educated, Industrialized, Rich, and Democratic) societies, yielding a field whose foundational constructs, measurement instruments, and intervention protocols reflect a particular cultural vantage point that may not straightforwardly generalize to the majority of the world's population (Henrich et al. 2010). Happiness is not experienced the same way everywhere; resilience does not draw on the same resources in every community; gratitude does not flow through the same relational channels across cultures. As positive psychology matures as a science, the systematic examination of how its core constructs operate—and how they must be adapted—in non‐WEIRD contexts has become not merely desirable but theoretically necessary.

This special issue takes up that challenge, with particular attention to the Chinese context and, more broadly, to non‐WEIRD societies across the Middle East and North Africa, Latin America, and non‐Confucian Asia. China represents an especially significant case for this inquiry. Its sociocultural fabric is woven from centuries of Confucian, Taoist, and Buddhist philosophical tradition, yielding value orientations—relational harmony, role‐based obligation, and collective interdependence—that resonate with some elements of positive psychology while sitting in productive tension with others (Joshanloo 2014). At the same time, China's extraordinary pace of economic development and urbanization has fundamentally reshaped the psychological landscape in which its citizens pursue well‐being, generating new pressures and new possibilities in education, work, family life, and aging (Mao, Peng, et al. 2022). These structural and cultural features make the Chinese context both a critical test case for the cross‐cultural validity of positive psychology and a generative source of new theoretical insight. Similar arguments apply, with appropriate contextual specificity, across other non‐WEIRD societies that remain substantially underrepresented in the literature, as evidenced by growing cross‐cultural research on institutional trust, civic behavior, and environmental responsibility spanning multiple nations (D'Ottone et al. 2025).

The core purpose of this special issue is threefold: to examine the cultural relevance and applicability of established positive psychology constructs in non‐WEIRD contexts; to explore the distinctive challenges and structural conditions that characterize these societies; and to draw out practical implications for intervention design, educational policy, workplace practice, and mental health promotion across diverse cultural settings. The issue is organized around five substantive themes, each addressing a domain where the gap between WEIRD‐based theory and non‐WEIRD lived experience is particularly consequential.

The first theme concerns well‐being and mental health in educational contexts. China's educational system subjects students and teachers alike to sustained academic pressure, high‐stakes examination cultures, and intense social comparison—conditions that standard Western models of academic well‐being are poorly equipped to capture. Research in this area has begun to demonstrate that positive psychology constructs such as flow experience, self‐efficacy, resilience, and positive relationships can be meaningfully mobilized in these high‐pressure environments, though the pathways through which they operate often reflect distinctively relational and culturally specific emphases (Jia et al. 2024; Mao, Xie, et al. 2022; Mao et al. 2024; Xie, Milani, et al. 2026). Papers invited under this theme are encouraged to examine how students and teachers in non‐WEIRD educational contexts navigate the intersection of achievement pressure and psychological flourishing, and to evaluate positive psychology interventions—such as strengths‐based development and meaning‐oriented approaches—when thoughtfully adapted to these settings.

The second theme addresses workplace well‐being and organizational applications of positive psychology. The Chinese workplace is shaped by distinctive structural and relational features—including Guanxi networks (Yang et al. 2021), hierarchical authority norms, and high performance expectations—that fundamentally condition how employees experience engagement, satisfaction, and flourishing. Accumulated research has demonstrated that constructs such as flow, psychological capital, authentic leadership, and strengths use operate through mechanisms that are both shaped by and reflective of these cultural and organizational conditions (Liu et al. 2023; Mao et al. 2023; Xie, Wang, et al. 2026). The challenge of adapting positive psychology interventions to diverse workplace populations also extends beyond the Chinese context: Effective interventions must move beyond group‐level cultural generalizations to address the personal relevance of the individual within their cultural context (Chandra and Leong 2016; Hall et al. 2021; Leong 2020). Papers invited under this theme are encouraged to examine how collective values and relational norms shape the conditions for flourishing at work, and how positive psychology interventions can be designed to be both culturally congruent and organizationally effective.

The third theme focuses on family dynamics and positive psychology. The family occupies a structurally and psychologically central position in Chinese and many other non‐WEIRD societies, functioning as the primary unit of social identity, obligation, and meaning‐making. Concepts such as filial piety, intergenerational reciprocity, and family harmony carry psychological weight with no direct parallel in Western positive psychology frameworks. Understanding how positive psychology constructs—including gratitude, positive parenting, relational well‐being, and life satisfaction—manifest within these family systems requires both cultural sensitivity and methodological rigor (Mao et al. 2015; Mao, Peng, et al. 2022; Pagliaro et al. 2021). Papers invited under this theme are encouraged to develop culturally informed accounts of how family relationships contribute to individual flourishing across the lifespan, with particular attention to structurally vulnerable populations such as left‐behind children and aging adults navigating shifting intergenerational obligations.

The fourth theme takes up mindfulness, resilience, and coping strategies. Mindfulness‐based interventions have become among the most widely disseminated tools in applied positive psychology, yet their conceptual origins in Buddhist contemplative tradition and their typical Western clinical packaging raise important questions about cultural fit when reintroduced into Asian contexts with their own living contemplative lineages. Similarly, resilience—understood as the capacity to adapt positively in the face of adversity—may draw on qualitatively different social and psychological resources in collectivist versus individualist cultural contexts, as research on the cooperative and relational dimensions of career adaptability in Chinese samples has begun to suggest (Mao et al. 2026; Prasad et al. 2021). Papers invited under this theme are encouraged to employ experimental, longitudinal, or network‐analytic designs to examine the mechanisms through which protective factors operate in non‐WEIRD contexts, moving beyond simple efficacy testing toward an understanding of why and for whom these approaches work.

The fifth theme addresses gratitude, meaning‐making, and positive aging. As China faces a profound demographic transition marked by rapid population aging, the psychological well‐being of older adults has become a matter of urgent social concern. Western models of successful aging tend to foreground personal autonomy and individual goal pursuit—values that may not map onto the relational and role‐based sources of meaning that characterize flourishing in later life for many Chinese older adults. Evidence suggests that gratitude, community engagement, place‐based belonging, and intergenerational connection function as distinctive pathways to well‐being in aging non‐WEIRD populations (Cai et al. 2024; Mao et al. 2015; Zhao et al. 2025). Papers invited under this theme are encouraged to examine how positive psychology interventions can be thoughtfully adapted to support flourishing in later life across diverse cultural contexts, and to consider how the relational and collective dimensions of aging in non‐WEIRD societies enrich and challenge existing theoretical frameworks.

Taken together, the contributions invited to this special issue respond to a fundamental challenge facing positive psychology as a global science: how to move beyond its WEIRD foundations without abandoning scientific rigor, and how to remain genuinely open to the possibility that engagement with cultural diversity will require not merely the adaptation of existing frameworks but their substantive theoretical enrichment. Cross‐cultural personality research has long demonstrated that constructs validated in Western samples may function differently in Chinese and other Asian populations, necessitating both measurement validation and theoretical revision (Cheung et al. 2011). For psychologists working across cultural contexts, an integrationist stance—neither wholesale adoption of Western frameworks nor retreat into cultural particularism—represents the most generative path for advancing both scientific rigor and cultural validity (Leong and Leung 2004). The Chinese context, with its deep philosophical traditions, rapid social transformation, and demographic scale, offers an exceptional opportunity for this kind of encounter between cultural specificity and scientific generalization. We hope the scholarship gathered in this issue will advance a more genuinely inclusive and culturally grounded positive psychology—one capable of contributing meaningfully to human flourishing in its full global diversity.

## Conflicts of Interest

The authors declare no conflicts of interest.

## Data Availability

Data sharing not applicable to this article as no datasets were generated or analysed during the current study.
